# Beauty Hinders Attention Switch in Change Detection: The Role of Facial Attractiveness and Distinctiveness

**DOI:** 10.1371/journal.pone.0032897

**Published:** 2012-02-29

**Authors:** Wenfeng Chen, Chang Hong Liu, Kazuyo Nakabayashi

**Affiliations:** 1 State Key Laboratory of Brain and Cognitive Science, Institute of Psychology, Chinese Academy of Sciences, Beijing, People's Republic of China; 2 Department of Psychology, University of Hull, Hull, England, United Kingdom; University of Sydney, Australia

## Abstract

**Background:**

Recent research has shown that the presence of a task-irrelevant attractive face can induce a transient diversion of attention from a perceptual task that requires covert deployment of attention to one of the two locations. However, it is not known whether this spontaneous appraisal for facial beauty also modulates attention in change detection among multiple locations, where a slower, and more controlled search process is simultaneously affected by the magnitude of a change and the facial distinctiveness. Using the flicker paradigm, this study examines how spontaneous appraisal for facial beauty affects the detection of identity change among multiple faces.

**Methodology/Principal Findings:**

Participants viewed a display consisting of two alternating frames of four faces separated by a blank frame. In half of the trials, one of the faces (target face) changed to a different person. The task of the participant was to indicate whether a change of face identity had occurred. The results showed that (1) observers were less efficient at detecting identity change among multiple attractive faces relative to unattractive faces when the target and distractor faces were not highly distinctive from one another; and (2) it is difficult to detect a change if the new face is similar to the old.

**Conclusions/Significance:**

The findings suggest that attractive faces may interfere with the attention-switch process in change detection. The results also show that attention in change detection was strongly modulated by physical similarity between the alternating faces. Although facial beauty is a powerful stimulus that has well-demonstrated priority, its influence on change detection is easily superseded by low-level image similarity. The visual system appears to take a different approach to facial beauty when a task requires resource-demanding feature comparisons.

## Introduction

The ability to detect changes in the natural environment is a fundamental skill for survival. However, detecting a change in rapidly alternating images separated by a blank mask (known as the flicker paradigm) can be extremely difficult (e.g., [1]). This poor performance in change detection is often referred to as “change blindness”. The flicker paradigm impairs change detection because the brief interval between the two images obscures the abrupt change caused by the discrepancy between the two images. Accumulated evidence suggests that attention can modulate change blindness (see [2] for a review). For example, detection is more likely when attention is directed to the object or location of the change. Thus, the ability to detect change is influenced by how easily attention is attracted to certain objects [2]. This suggests the possibility that a change occurred on certain important stimuli (e.g., faces) can be detected more efficiently.

Research has shown that faces can attract more attention compared to other generic objects. For example, Hershler and Hochstein [3] found that detection of human faces among a variety of objects in a visual search task is nearly independent of the size of the search array. In other words, a human face appears to ‘pop out’ when it is shown among other objects although faces among themselves are generally processed in a serial manner [4]. Using eye movement measures, Theeuwes and van der Stigchel [5] observed delayed saccadic response to locations that previously contained a face. These results provide converging support for the notion that faces may have a special capacity to summon and recruit attention when they compete with other environmental stimuli for attention. To test the same hypothesis, Ro, Russell and Lavie [6] used the flickering paradigm where participants had to detect a change that either occurred on a face or on one of five other common objects. They found that changes to faces were detected more efficiently than changes to objects.

Apart from competing with other environmental stimuli, faces can also compete with each other for attention. It is well known that an angry or fearful face can attract attention more easily when it is shown among faces with a neutral expression. Attention to these important emotional signals is often rapid, unconscious and mandatory (see [7], for a review). However, Ohman, Lundqvist, and Estevesas [8] have also shown that the angry face advantage could be reduced if emotional faces rather than neutral faces were used as distractors. This result was originally demonstrated with schematic faces. Recently it has also been replicated with more realistic photographic stimuli [9], [10]. These studies suggest that certain emotional expressions can redirect attention away from a task-relevant target. This could lead to less efficient processing of the target.

In this study, we examine whether similar effects could be found with a different kind of facial information—facial attractiveness. Our purpose was to investigate how spontaneous appraisal of facial beauty affects attention in change detection. Like facial expression, facial beauty has also been found to attract more attention because of its important social and biological implications. Research has shown that people tend to look longer at attractive than at unattractive faces [11]. Facial beauty can be appraised automatically and rapidly [12]. Beautiful faces may capture attention even when they are shown outside the foveal vision. Using a spatial cuing task, Sui and Liu [13] found that a laterally presented, task-irrelevant beautiful face would automatically compete with an ongoing cognitive task for spatial attention.

Although facial attractiveness is known to modulate attention, no study to date has examined how it affects distribution of attention among multiple faces. Complex demands could arise with the presence of multiple faces. For example, selective attention could be less focused for multiple attractive faces than for a single attractive face. Furthermore, the effect of facial attractiveness on attention has only been found in a simple perceptual task with brief presentation [13]. It is not known whether facial attractiveness also modulates attention in a task where a much slower, laborious, and more controlled serial search process is involved, such as a change detection task. We chose change detection as our experimental paradigm, because it taps into the effortful attentional mechanisms by which the central executive assigns priority in response to task requirements. Prior research has mainly looked into the effect of task-irrelevant facial beauty on a more transient and reflexive aspect of the attentional system. A key aim of this study was to explore how different compositions of attractive and unattractive faces on a display affect change detection where appraisal of facial attractiveness is task irrelevant. Using the flicker paradigm in which detection of facial identity change is either made among multiple attractive faces or multiple unattractive faces, we aimed to examine whether the presence of attractive/unattractive distractors affects change detection performance. Prior research suggests that the detection advantage for faces relative to other objects in a change detection task can be attenuated or disappear when multiple faces are introduced into a display [14]. This evidence suggests that distributing attention among multiple faces could affect change detection performance. We hypothesized that, relative to unattractive distractors, the presence of attractive distractors would create a stronger interference in a change detection task because they may hold attention away from the change location.

However, existing evidence suggests that detection performance in this task could also be influenced by facial distinctiveness. Distinctiveness is often defined by a marked deviation from a population mean. In contrast to distinctive faces, typical faces are judged as more similar to other faces [15]. Because of this, inter-item similarity among faces could be used to measure typicality or distinctiveness. It has been shown in the change detection literature that a change on a distinctive face is detected more quickly than on a typical face [16]. It is also known that unattractive faces are generally more distinctive than attractive faces [17]. Attractiveness ratings have been found to correlate negatively with distinctiveness ratings [18]. Based on these findings, distinctiveness and attractiveness may have opposite effects on change detection. Another determinant of detection performance is the magnitude of the change. A larger change is known to alleviate change blindness [19], [20]. Moreover, a high degree of visual similarity between the pre- and post-change targets can counteract the face-capturing effect in change detection [14].

Given these findings, the detection of identity change may be determined by several factors including attractiveness, distinctiveness, as well as the magnitude of image difference between the faces that are involved in an identity change. Hence, another key aim of this study was to determine how spontaneous appraisal for facial beauty interacts with other lower level visual analyses in change detection. Two experiments were designed to identify the contribution of these factors to the distribution of attention.

The purpose of Experiment 1 was to investigate whether multiple attractive faces affect change detection when facial distinctiveness was taken into account. Based on prior research, both attractiveness and distinctiveness were expected to affect detection performance. We attempted to control the influence of the two factors by measuring the effect of attractiveness when the level of distinctiveness was equalized. Following [15], we used inter-item similarity as a measure of facial distinctiveness. However, instead of subjective rating, we adopted an objective similarity measure in this experiment. Image similarity between target and distractor faces in each trial was determined by the Structural SIMilarity Index (SSIM). Developed by Wang, Bovik, Sheikh, and Simoncelli [21], this similarity measure employs global structural information of images. It evaluates structural changes between two complex-structured signals to take into account the perceived changes in structural information variation. The SSIM output ranges from -1 (entirely different) to 1 (identical). SSIM was adopted because it was an improved measure over the traditional measurements of similarity such as peak signal-to-noise ratio (PSNR) and mean squared error (MSE). Moreover, SSIM is also more consistent with human perception [21]. A Matlab implementation of the SSIM index (ssim_index.m) is available at https://ece.uwaterloo.ca/~z70wang/research/ssim/. Using this method, we calculated the SSIM scores for the two alternating targets and the three distractors in each change-present condition. We then took the mean scores between the target and distractor faces as the measure of distinctiveness. Given that multiple attractive faces could create additive demands for attention, we expected poorer change detection performance for this condition than for the condition that consisted of only unattractive stimuli. This prediction is based on the fact that it is more difficult to disengage attention from an attractive face [22]. To perform the detection task effectively, it is necessary to switch and disengage attention rapidly from one face to the next. When all faces on a display are attractive, every face could contribute to the delay of this attention-switching process because they could all require more time for inspection. In addition, we predicted poorer change detection performance for less distinctive stimuli.

Because all faces in Experiment 1 were either attractive or unattractive, the automatic appraisal of facial attractiveness could only have an interference effect on detection. To explore whether such task-irrelevant appraisal of facial beauty could also facilitate change detection, Experiment 2 employed a condition where an attractive target was shown among multiple unattractive distractors. Because no other faces could compete with the target's level of attractiveness for attention in this condition, detection of an identity change on the attractive target should be carried out more efficiently than the second new condition where an unattractive target was shown among multiple attractive distractors. Experiment 2 also further investigated the effect of inter-item similarity. Although image difference between target and distractor faces was controlled in Experiment 1, the effect of the magnitude of change between the alternating target faces was not examined. Because the magnitude of change between the two alternating target images may also be affected by subjective impressions, we asked participants to judge how different the alternating target faces were after they had completed the change detection task in Experiment 2. We then examined the extent to which their change detection performance was correlated with their similarity judgments. If the magnitude of change plays a more important role than attractiveness in change detection, then detection performance should be mainly determined by the magnitude of change than by level of attractiveness. If the magnitude of change is comparable in attractive and unattractive pairs, then attractive targets should be detected better. Because Experiment 1 only showed an effect of multiple attractive faces for low distinctiveness condition, the high distinctiveness condition was excluded in Experiment 2.

## Results

### Experiment 1

**Figure 1:**
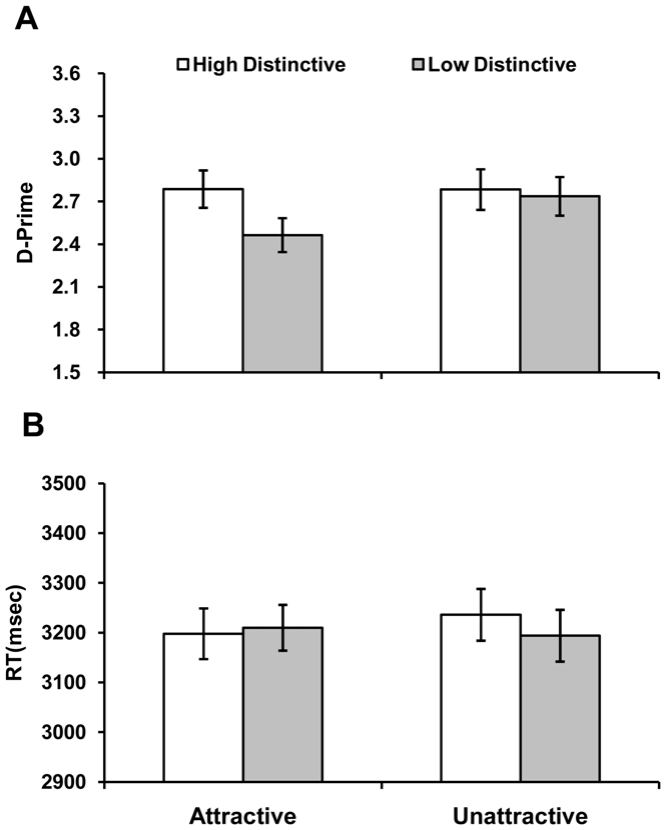
Mean percent accuracy and reaction time as a function of distinctiveness and attractiveness. Error bars represent one standard error about the mean.

The *d′* was calculated for each participant. The log-linear rule was used to correct for extreme hit rate and false alarm rate before the calculation [23]. The *d′* and RT results are shown in Figure 1. The criterion results are shown in Table 1. These data were analyzed using repeated-measures analyses of variance (ANOVAs).

**Table 1:** Mean criterion results in Experiment 1 (Values in parentheses represent standard deviations).

Distinctiveness	Attractiveness	
	Attractive	Unattractive
High	0.68(0.28)	0.76(0.30)
Low	0.84(0.35)	0.72(0.29)

The *d′* results showed nearly significant or significant main effects of attractiveness, *F* (1, 34) = 3.68, *p* = .06, and distinctiveness, *F* (1, 34) = 7.00, *p*<.05. The interaction between these was also significant, *F* (1, 34) = 5.62, *p*<.05. Simple main effect analyses showed a better detection performance for unattractive faces relative to attractive faces when faces were undistinctive, *F* (1, 34) = 7.53, *p*<.05. However, when faces were distinctive, there was no difference between the results of attractive and unattractive faces, *F* (1, 34)<0.01, *p* = .97. It was also found that distinctive faces were better detected than undistinctive faces when the faces were attractive, *F* (1, 34) = 15.95, *p*<.01. However, there was no significant difference between high and low distinctiveness when the faces were unattractive, *F* (1, 34) = 0.23, *p* = .64.

The criterion results showed no main effects of attractiveness, *F* (1, 34) = .36, *p* = .55, or distinctiveness, *F* (1, 34) = 2.30, *p* = .14. However, there was a significant interaction, *F* (1, 34) = 10.01, *p*<.01. Simple main effect analysis showed that undistinctive faces resulted in a more conservative criterion than distinctive faces when the faces were attractive, *F* (1, 34) = 8.70, *p*<.01. However, there was no significant difference between the two levels of distinctiveness when the faces were unattractive, *F* (1, 34) = 0.59, *p* = .45.

The RT data showed no main effects or interaction, *Fs* (1, 34)<1, *ps*>.35. To investigate whether our results were affected by a speed-accuracy tradeoff, we conducted a median-split analysis following the method in Hein, Rolk, and Ulrich (2006) [24]. To separate detection performance for short and long RTs, we first computed a median RT for each participant in each condition. The trials in each condition were then sorted into shorter or longer RTs according to the median. ANOVA with this additional factor of response speed (short vs. long RTs) replicated the analysis without the split: the main effects of attractiveness and distinctiveness and the interaction between the two factors were not significant for the RT data, *Fs* (1, 34)<2.30, *ps*>.14. Critically, response speed did not interact with attractiveness or distinctiveness, *F*s (1, 34)<2.28, *p*s>.14. This suggests that the effects in this experiment were not contaminated by a speed-accuracy tradeoff.

### Experiment 2

**Figure 2:**
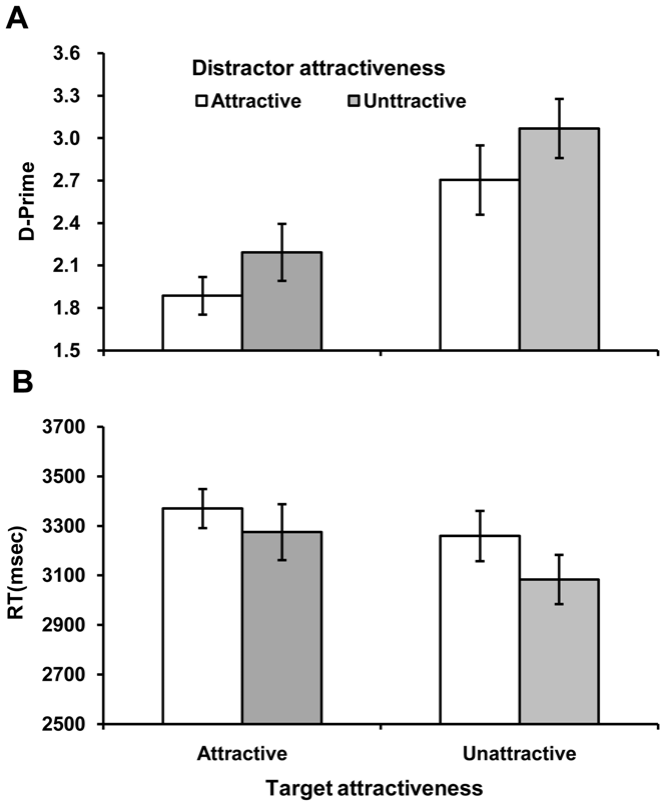
Mean percent accuracy and reaction time as a function of attractiveness. Error bars represent one standard error about the mean.

Results of *d′* and response time are shown in Figure 2. Results of criterion are shown in Table 2. The *d′* results showed significant main effects for both target and distractor attractiveness. Detection performance was more accurate when unattractive faces were used as targets, *F* (1, 27) = 19.66, *p*<.01. Unattractive faces also enjoyed advantage relative to attractive faces when they were used as distractors, *F* (1, 27) = 3.74, *p* = .06. The interaction between the two factors was not significant, *F* (1, 27) = .06, *p* = .81.

**Table 2:** Mean criterion results in Experiment 2 (Values in parentheses represent standard deviations).

Distractors	Target	
	Attractive	Unattractive
Attractive	0.33(0.38)	0.07(0.46)
Unattractive	0.36(0.57)	0.07(0.62)

The criterion results showed a significant main effects for target attractiveness, *F* (1, 27) = 9.92, *p*<.01, where response criterion was more conservative for attractive faces than for unattractive faces. The main effect of distractor attractiveness or the interaction between the two factors was not significant, *Fs* (1, 27)<0.06, *ps*>.82.

The RTs results showed that attractive targets were detected more slowly than unattractive targets, *F* (1, 27) = 8.70, *p*<.01. The detection was also slower when the target was shown among attractive distractors than among unattractive distractors, *F* (1, 27) = 7.77, *p*<.05. The interaction between target and distractor attractiveness was not significant, *F* (1, 27) = .21, *p* = .65. An evaluation of speed-accuracy tradeoff similar to Experiment 1 replicated results of the above ANOVA: significant main effects were found for both target attractiveness, *F* (1, 27) = 13.20, *p*<.01, and distractor attractiveness, *F* (1, 27) = 10.70, *p*<.01, and the interaction between the two was not significant, *F* (1, 27) = 1.29, *p* = .27. Importantly, response speed did not interact with target attractiveness and distractor attractiveness, *F*s (1, 28)<2.08, *p*s>.16. This suggested that the effects in this experiment were not affected by speed-accuracy tradeoff.

The rating data revealed that participants found the alternating target images less distinguishable from each other when the faces were attractive: The mean rating scores were 3.86 (*SD* = 1.25) for attractive pairs and 4. 39 (*SD* = 1.45) for unattractive pairs. A one-way ANOVA showed that the attractive face pairs were more physically similar than the unattractive face pairs, *F* (1, 813) = 31.73, *p*<.001. We also used SSIM to measure the magnitude of change. The mean SSIM scores were 0.76 (*SD* = 0.04) for attractive face pairs and 0.72 (*SD* = 0.04) for unattractive face pairs. Similar to the rating data, the physical similarity between the target faces in unattractive pair was again significantly smaller than attractive face pairs, *F* (1, 813) = 172.25, *p*<.001.

Based on the rating data and SSIM scores, we performed a Pearson correlation analyses between the measures of change magnitude and detection performance. The results showed that the detection accuracy was significantly correlated with both of these measures (*r*s = −0.18 and 0.20, *p*s<.001), and the detection RT was also significantly correlated with both of these measures (*r*s = −0.16 and 0.15, *p*s<.001). This suggests that the participants' ratings and SSIM both measured magnitude of change, such that physical distinctiveness led to better detection performance. There was also a significant correlation between the participants' ratings and the SSIM scores (*r* = −0.29, *p*<.001), suggesting that participants' ratings were to some extent based on physical distinctiveness.

However, could the results of correlation between change magnitude and detection performance mean that the effects in this experiment were solely due to the magnitude of change or physical similarity? To address this question, we conducted an item-based ANCOVA analysis where faces were treated as a random factor, target and distracter attractiveness as independent variables, and inter-item similarity and change magnitude as covariates. This allowed us to assess whether attractiveness also had an effect on performance after removing the covariates. ANCOVA on the accuracy data showed comparable performance for attractive and unattractive faces as target stimuli, *F* (1, 90) = 1.89, *p* = .17. However, when used as distractors, unattractive faces produced a marginally significant advantage relative to attractive faces, *F* (1, 90) = 3.09, *p* = .08. The interaction between the two factors was not significant, *F* (1, 90) = .46, *p* = .50. Performance was also significantly affected by change magnitude, *F* (1, 90) = 21.32, *p*<.001, as well as by inter-item similarity, *F* (1, 90) = 8.55, *p*<.01. ANCOVA on the RT data showed that attractive targets were detected more slowly than unattractive targets, *F* (1, 90) = 3.08, *p* = .08. Change detection was also slower when the target was shown among attractive distractors than among unattractive distractors, *F* (1, 90) = 2.89, *p* = .09. The interaction between target and distractor attractiveness was not significant, *F* (1, 90) = .73, *p* = .39. Finally, response time was also significantly affected by change magnitude, *F* (1, 90) = 13.35, *p*<.001, but not by inter-item similarity, *F* (1, 90)<.01, *p*>.98. In sum, these analyses show that although similarity alone can account for the effect of target on accuracy, it cannot account for the effect of target on RT by itself. Moreover, neither the accuracy nor RT effects for distractor attractiveness can be accounted for by similarity alone.

## Discussion

Experiment 1 showed that the participants' ability to detect an identity change was significantly impaired when all faces in a trial were attractive. However, this effect was only found for the low distinctiveness condition where image difference between target and distractor faces was small. The effect of attractiveness confirms our hypothesis that the presence of multiple attractive faces may disrupt effective distribution of attention. However, when target and distractor faces are distinct from each other, the benefit from this information appears to reduce the detrimental effect of attractiveness on change detection. This suggests that the effect of attractiveness in change detection is modulated by distinctiveness.

Experiment 1 also showed that distinctiveness had no significant effect on change detection when all faces in a trial were unattractive. This result suggests that the benefit of distinctiveness is quite negligible in this condition. However, when attractive faces are processed at the same time, the benefit of distinctiveness could become more visible.

Experiment 2 showed that detection of identity change was poorer when attractive faces were present in a trial. This effect was present no matter whether the target faces were attractive or unattractive. Subjective rating and objective SSIM scores have yielded consistent measures of change magnitude for the alternating targets. Analyses based on both measures showed that the change between unattractive faces in a target pair was greater than in an attractive target pair. Moreover, consistent with Yang et al. (2009) [14], our data suggest that smaller magnitude of change is correlated with poorer change detection performance. This result suggests that detection of identity change is influenced by the similarity between alternating faces. Facial attractiveness itself may play unimportant role in change detection.

The results from both experiments suggest that change detection performance may be inversely related to the number of attractive faces used in a trial. The poorer performance in the attractive condition of Experiment 1 may be due to greater difficulty to disengage attention from attractive distractors. This is consistent with the interpretation of [22], who demonstrated a similar effect with a single attractive face in a dot probe task. This simple explanation appears to be consistent with some of our data in Experiment 2. Figure 2 shows that the best detection performance in this experiment was found in the condition where not a single face was attractive in a trial, followed by the conditions where either target or distractor faces were attractive. The poorest detection was found when all faces were attractive. These results suggest that participants could be inadvertently delayed by paying more attention to attractive faces even though appraisal of attractiveness was task-irrelevant. The distraction effects in our study may resemble other effects of face stimuli found in visual search tasks, where emotional distractor faces are found to produce poor search performance [8]–[10]. It has been argued that a change detection task is analogous to a serial search task in that both require effective shift of attention from one stimulus item to another [19], [20].

Because participants were unaware of the purpose of this research, the effects of facial attractiveness on change detection may reflect an automatic appraisal for facial attractiveness. The results from the present study show that this involuntary appraisal not only affects transient attentional capture in a covert attention task [13], but also modulates attention in a change detection task, where attention is guided by a slow, controlled, serial search process [19], [25]. However, the present study also shows that a resource-demanding attention task can produce very different effects of facial attractiveness. The attractiveness effect is more easily superseded by low-level image similarity in this task.

A rather surprising and puzzling finding in Experiment 2 is that when an attractive target was shown among unattractive distractors, the detection performance was not better than an unattractive target being shown among attractive distractors. If all distractors are unattractive, attention should be more easily switched to the attractive target. By the same logic, it would be more difficult to pay attention to an unattractive target if attention is engaged on attractive distractors. This result is also detrimental to the conjecture that it is more difficult to disengage attention from attractive faces. Although an exact explanation for this result is yet to be found, it is possible that the physical difference between the alternating face stimuli on change detection played a more decisive role in these conditions. Much research has suggested that low level stimulus properties play an important role in change detection [14], [16], [26]. Consistent with prior observations, the data in both of our experiments showed a great impact of image similarity. Experiment 1 showed that the detrimental effect of attractive faces was bigger in trials with undistinctive faces, and possibly the effect does not occur when faces are distinctive. Experiment 2 revealed a clear correlation between detection performance and the magnitude of change occurred to the target face. However, our manipulation and analysis suggest that the results in this study cannot be explained by similarity alone. To find out whether attractiveness affects change detection, we matched similarity/distinctiveness between the attractive and unattractive stimuli. In Experiment 1, both attractive and unattractive stimuli had SSIM scores of 0.70 for high distinctiveness and 0.74 for low distinctiveness. Hence it would be difficult to explain the effects of attractiveness in Experiment 1 based on similarity alone. In Experiment 2, we only used faces of low distinctiveness. Again, we matched the target and distractor similarity for attractive and unattractive conditions. However, because the similarity between the alternating targets (change magnitude) was not matched in this experiment, we conducted an ANCOVA analysis that allowed us to evaluate whether attractiveness produced an effect on the detection performance after removing the effect of similarity. The results show that similarity alone can only account for the effect of target attractiveness on accuracy. It cannot account for the effect of target attractiveness on RT. Moreover, neither the accuracy nor the RT results for distractor attractiveness can be accounted for by similarity alone.

There is evidence that high level salience can dominate low level salience in change detection. For example, a change in scene-inconsistent objects was detected more quickly and accurately than in scene-consistent objects for both high and low visually salient objects [16]. In contrast to this, the high level salience due to attractiveness could not outweigh the impact of low level physical similarity in our study. Consistent with prior research [13], the effect of task-irrelevant attractiveness on attention is small and the effect only was only significant under restricted conditions where target and distractor faces were not distinct from each other. This suggests that observers are able to maximize performance by suppressing task-irrelevant activities although this may not completely abolish the spontaneous tendency to appraise faces for attractiveness.

In summary, the presence of multiple attractive faces may impair the detection of target identity change. Although the effect of facial attractiveness can be demonstrated in a change detection task, it may only manifest itself when low-level cues in terms of image similarity are not readily available. On the other hand, the magnitude of change is proven a reliable predictor for detection performance. An identity switch between two attractive faces is more difficult to detect relative to two unattractive faces because attractive faces are often more difficult to distinguish from each other.

## Materials and Methods

### Participants

The study was approved by the ethics committee of the Psychology Department in the University of Hull. Written consent was acquired from each participant prior to the experiment. Participants were undergraduate students. Thirty five of them (22 females and 13 males, age: *M* = 22.1, *SD* = 5.4) participated in Experiment 1 and 28 (20 females and 8 males, age: *M* = 19.8, *SD* = 2.4) participated in Experiment 2. All participants had normal or corrected-to-normal vision.

### Stimuli

The face database was obtained from the University of St. Andrews. It contains frontal-view Caucasian faces with no external features (hair and clothing). All faces in the database were rated by 19 raters (aged between 18 and 29 years, 12 females) for attractiveness on a 7-point scale. Two sets of female faces (one attractive, the other unattractive) were selected. The mean attractiveness ratings for the two sets were 4.47 (*SD* = 0.35, *N* = 24) and 2.04 (*SD* = 0.26, *N* = 24) respectively. All faces were cropped with same-size oval shape and the size was normalized according to the face width. The resulting image measured 4.7×6.1 cm (4.4×5.8°) on screen. All images were scaled to the same mean luminance and root-mean-square contrast.

In each change-present trial, five faces were randomly chosen from the two image sets. Two of them served as the changing target and the remaining three as the unchanged distractors. The image difference between the target and distractor faces in each trial was classified as high or low according to the SSIM scores of the five images. In the low image difference category, there were 84 pairs of attractive and 75 pairs of unattractive faces. In the high image difference category, there were 85 pairs of attractive and for 89 of unattractive faces. The SSIM scores for these pairs in the two categories were significantly different from each other (0.70 vs. 0.74), *t* (331) = 23.40, *p*<.001. Faces were carefully selected such that the distinctiveness between the attractive and unattractive stimuli was comparable (0.70 for attractive face pair with high distinctiveness, 0.70 for unattractive face pair with high distinctiveness; 0.74 for attractive face pair with low distinctiveness, and 0.74 for unattractive face pair with low distinctiveness).

The stimuli were displayed on a 21″ monitor (SONY Trinitron, GDM-F520). The background color of the display was black. E-Prime (Version 1.2) was used to generate the dynamic alternation of stimuli and to control the flow of the experiment. Experiments 1 and 2 used the same stimuli except that only the faces used in the low distinctiveness condition were included in Experiment 2.

### Design

We employed a within-participant design. In Experiment 1, the independent variables were attractiveness (attractive vs. unattractive) and distinctiveness (high vs. low). The faces in each trial were either all attractive or all unattractive. In Experiment 2, the independent variable was the target attractiveness (attractive vs. unattractive) and distractor attractiveness (attractive vs. unattractive).

### Procedure

**Figure 3:**
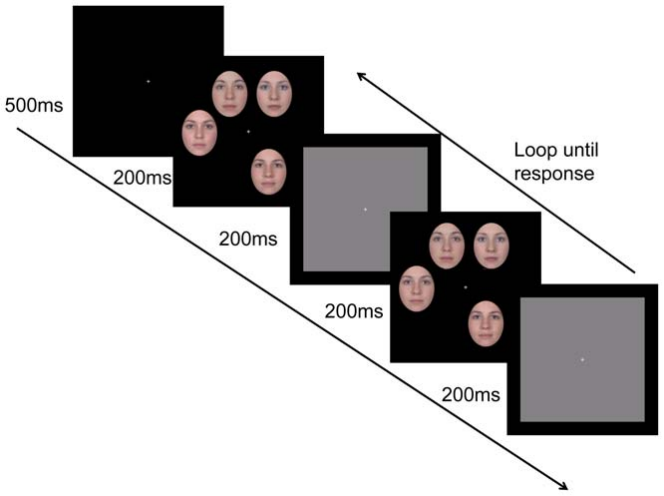
Illustration of the procedure in the study. The faces in this figure are morphed to protect the identity of individuals.

Participants were tested individually. An adjustable headrest was used to fix the participant's viewing position, which was set 60 cm away from the computer monitor. In each trial, faces appeared randomly in four of six place holders on an imaginary circle (6.4 degrees of radius from the central fixation), with the constraint that the faces must occupy two place holders on each of the left and right half of the circular array. Overall, a change of face identity occurred in these locations with equal probability. Each trial consisted of two alternating frames of four faces (see Figure 3 for an illustration). The position of the faces in the two frames was identical. Both frames were shown for 200 ms with a blank frame of 200 ms inserted in between. This sequence was looped until the participant pressed one of two keys, indicating whether or not a change of identity had occurred to one of the faces. In the change-absent trials, the two frames consisted of identical faces. In the change-present trials, one of the four faces in the first frame was a different identity from the face of the correspondent location in the second frame. The order of the change-present and change-absent trials was random, with the constraint that no more than three consecutive change present/absent trials could happen in a row.

In Experiment 1, there were 3 blocks of 60 trials following a 20-trial practice session. After each block participants were given feedback for their response accuracy and given the opportunity to take a short break. Each of the four conditions (2 attractiveness×2 distinctiveness) had 45 trials. On average, participants took 20 minutes to complete Experiment 1.

In Experiment 2, as well as the trials where all faces were either attractive or unattractive, we also included trials where attractive distractors were shown with an unattractive target, or unattractive distractors were shown with an attractive target. The target always consisted of faces of comparable attractiveness. There were four blocks of 70 trials after 20 practice trials. After the change detection task, the target face pairs were presented side by side, one pair at a time, on the screen. Participants were instructed to rate how distinguishable the pair of faces were from each other on a 7-point scale, where 1 indicated very difficult and 7 indicated very easy to distinguish. On average, it took participants 45 minutes to complete Experiment 2.
